# Complement Factor H-Related Protein 3 Serum Levels Are Low Compared to Factor H and Mainly Determined by Gene Copy Number Variation in *CFHR3*

**DOI:** 10.1371/journal.pone.0152164

**Published:** 2016-03-23

**Authors:** Richard B. Pouw, Mieke C. Brouwer, Judy Geissler, Laurens V. van Herpen, Sacha S. Zeerleder, Walter A. Wuillemin, Diana Wouters, Taco W. Kuijpers

**Affiliations:** 1 Department of Immunopathology, Sanquin Research and Landsteiner laboratory of the Academic Medical Center, University of Amsterdam, Amsterdam, the Netherlands; 2 Department of Pediatric Hematology, Immunology & Infectious Diseases, Emma Children’s Hospital, Academic Medical Center, Amsterdam, the Netherlands; 3 Department of Blood Cell Research, Sanquin Research and Landsteiner Laboratory of the Academic Medical Center, University of Amsterdam, Amsterdam, the Netherlands; 4 Department of Hematology, Academic Medical Center, University of Amsterdam, Amsterdam, the Netherlands; 5 Division of Hematology and Central Hematology Laboratory, Luzerner Kantonsspital and University of Berne, Berne, Switzerland; Centre for Eye Research Australia, AUSTRALIA

## Abstract

The major human complement regulator in blood, complement factor H (FH), has several closely related proteins, called FH-related (FHR) proteins. As all FHRs lack relevant complement regulatory activity, their physiological role is not well understood. FHR protein 3 (FHR-3) has been suggested to compete with FH for binding to *Neisseria meningitidis*, thereby affecting complement-mediated clearance. Clearly, the *in vivo* outcome of such competition greatly depends on the FH and FHR-3 concentrations. While FH levels have been established, accurate FHR-3 levels were never unequivocally reported to date. Moreover, *CFHR3* gene copy numbers commonly vary, which may impact the FHR-3 concentration. Hence, we generated five anti-FHR-3 mAbs to specifically measure FHR-3 in human healthy donors of which we determined the gene copy number variation at the *CFH/CFHR* locus. Finally, we examined the acute-phase response characteristics of FHR-3 in a small sepsis cohort. We determined FHR-3 levels to have a mean of 19 nM and that under normal conditions the copy number of *CFHR3* correlates to a very large extent with the FHR-3 serum levels. On average, FHR-3 was 132-fold lower compared to the FH concentration in the same serum samples and FHR-3 did not behave as a major acute phase response protein.

## Introduction

Complement factor H (FH) is the major regulator of the complement activation cascades in blood, being produced in the liver and circulating at a concentration of approximately 2 μM [1–4]. Next to FH, humans possess several closely related proteins of which the function is still unclear because of the lack of appropriate tools for their accurate detection and functional testing. Together these proteins form the FH protein family, comprising FH, its splice variant FH-like 1, and 6 FH-related (FHR) proteins, numbered 1 to 5 including the splice variants 4A and 4B. The genes of the FH protein family are located in tandem on chromosome 1q31 in the following order: *CFH*, *CFHR3*, *CFHR1*, *CFHR4*, *CFHR2* and *CFHR5*.

The FHR proteins show a remarkable sequence similarity to FH and all consist entirely of so-called short consensus repeat (SCR) domains, each of approximately 60 amino acids. Recent interest in FHR-3 stems from the association with meningococcal disease [5] and its potential to interfere with FH binding to the meningococcal capsular protein, FH-binding protein (fHbp). By hijacking FH, fHbp protects the meningococcus from *in vitro* complement-mediated lysis, which could be countered by FHR-3 (unpublished observations) [6,7]. FHR-3 has 5 SCR domains, each with a striking sequence identity with SCR domains of the complement regulator FH and other FHR proteins, in particular with FHR-4A/B [8]. The reported molecular weight of serum-derived FHR-3 ranges from 37 to 50 kDa due to different glycosylation variants [8]. Together with FHR-4A/B, FHR-3 forms a subgroup of closely related FHRs, whereas FHR-1, FHR-2 and FHR-5 form a subgroup that is characterized by a dimerization motif in the first two SCR domains resulting in homo- and hetero-dimerization, which is not present in FHR-3 and FHR-4A/B [9].

*CFHR3* is commonly deleted due to homologous recombination–most often together with *CFHR1*. This deletion is reported to occur in about 15% of healthy individuals with ethnic variations in its prevalence [10,11]. *CFHR3/CFHR1* deletion is associated with a decreased risk for the development of age-related macular degeneration (AMD) on the one hand, as well as with an increased risk for the development of atypical hemolytic uremic syndrome (aHUS), which, in the case of aHUS, seems to be partly explained by the appearance of anti-FH auto-antibodies [12–14].

FHR-3 has been reported to directly act as a complement regulator due to exhibiting weak co-factor activity for complement factor I, resulting in degradation of C3b [15]. In addition, FHR-3 can directly bind C3b via a seemingly similar mechanism as FH [15,16]. Currently, FHR-3 is hypothesized to act as a “de-regulator” of the complement system through competition between FH and FHR-3 for the binding of either C3b or host surfaces, thus enhancing the complement activation in a positive manner [17]. This is explained by the fact that FHR-3, like all FHRs, lacks any SCR domains identical to N-terminal SCR domains of FH reported to regulate C3, while SCR domains identical to SCRs of FH associated with C3b and host surface binding are present. The “de-regulator” hypothesis might also explain the association of *CFHR3/CFHR1* deficiency with a decreased risk for AMD, as a lack of FHR-3 would thus allow for better surface binding and consequently complement regulation by FH [17].

Recently, Caesar *et al*. (2015) reported the binding of FHR-3 to meningococcal fHbp *in vitro*, resulting in decreased survival of *Neisseria meningitidis* in human serum [7], although such competition on bacterial surfaces *in vivo* will strongly depend on the blood levels of both proteins. Whereas FH serum levels have been established with an average concentration of approximately 2 μM [1–4], FHR-3 serum levels have only been estimated to circulate at a similar molar concentration, but without accurate measurement due to the lack of specific reagents for accurate and reliable quantification [15]. Measuring FHR proteins remains challenging due to the high degree of sequence identity between the FHR proteins as well as with FH. In this study we report a FHR-3-specific ELISA with the use of monoclonal antibodies (mAbs) to establish normal serum levels, describe the influence of copy number variation (CNV) in healthy donors in relation to FHR-3 levels and finally, investigate the acute-phase characteristics of FHR-3 in sepsis.

## Material and Methods

### Samples

Blood samples were drawn from anonymous, healthy volunteers with informed, written consent in accordance with Dutch regulations and this study was approved by the Sanquin Ethical Advisory Board in accordance with the Declaration of Helsinki. Serum was obtained by collecting blood, allowing it to clot for 1 hour at room temperature (RT) and collecting the supernatant after centrifugation at 3000 rpm for 10 min. Plasma and peripheral blood leukocytes were collected from EDTA blood samples and subsequently used for DNA extraction using the QIAamp DNA Blood Mini Kit (Qiagen, Hilden, Germany) according to the manufacturer’s instructions. All samples were stored in small aliquots at -80°C until use to avoid repetitive freeze/thawing. Serum samples of septic patients were collected as part of a previous study with informed consent according to the local ethics committee in accordance with the Declaration of Helsinki [18]. Only samples taken upon inclusion into the original study (n = 39) were used in this study. Patient characteristics and CRP levels were determined and reported as part of the original study [18].

### Proteins and monoclonal antibodies

Rat anti-mouse kappa (RM-19) mAb was from Sanquin Business Unit Reagents (Sanquin, Amsterdam, the Netherlands). Mouse mAbs directed against human FH SCR domains 16/17 (anti-FH.16) were obtained as part of another study at our laboratory (manuscript in preparation). Polyclonal goat anti-human FH was obtained from Quidel (San Diego, CA, USA) and conjugated with HRP. High Performance ELISA buffer (HPE) was provided by Sanquin. Proteins were biotinylated according to the manufacturer’s instructions using EZ-Link Sulfo-NHS-LC-Biotin, No-Weigh Format (Thermo Scientific), when indicated.

### Expression of FHR proteins

Constructs containing the cDNA sequences of all FHR proteins, including both known splice variants of *CFHR4*, were ordered at Invitrogen (Paisley, UK). Constructs were ordered with an in-frame C-terminal tag coding for six histidine residues (6xHis) and cloned into pcDNA 3.1 vectors (Invitrogen). Proteins were transiently expressed in HEK293F cells with 293Fectin and OptiMEM (Invitrogen), using the Freestyle HEK293F expression system (Invitrogen) according to the manufacturer’s instructions. Five days after transfection, supernatants were collected and recombinant human (rh) FHR proteins were purified by Ni^2+^ affinity chromatography with the use of HisTrap High Performance 1 ml columns (GE Healthcare Life Sciences, Freiburg, Germany) according to the manufacturer’s instructions. Subsequent filtrations using Amicon Ultra Centrifugal Filter Devices (Merck Millipore, Darmstadt, Germany) were performed to obtain highly pure rhFHR-3. Throughout the purification process, purity of the rhFHR proteins was assessed by SDS-PAGE under non-reducing conditions and visualized by silver staining using the SilverQuest Staining kit (Invitrogen) according to the manufacturer’s instructions.

### Immunization and hybridoma generation

Polyclonal anti-FHR-3 antibodies were obtained by immunizing a rabbit via i.m. injection with 100 μg rhFHR-3 in montanide as adjuvant at four week intervals. Three days after the fourth booster, an IgG fraction was obtained from serum with the use of a ProtG sepharose column.

Anti-FHR-3 mAbs were generated by immunizing BALB/c mice via i.p. injection with 25 μg rhFHR-3 in montanide at four week intervals. Three days after the fourth booster, spleen cells were isolated and fused with the mouse myeloma cell line SP2/0. The presence of FHR-3 specific antibodies in the supernatants of the hybridomas was tested by ELISA. In short, Nunc Maxisorp 96-wells microtiter plates (Invitrogen) were coated overnight at room temperature with 1 μg/ml RM-19 mAb in PBS to capture mouse kappa immunoglobulins from the supernatants. After coating, plates were washed five times with PBS + 0.02% (v/v) Tween-20 (PT), followed by incubation with 25% (v/v) supernatant, diluted in HPE, for 1 h. After washing, specificity of bound antibodies was determined by addition of 1 μg/ml biotinylated rhFHR-3 (rhFHR-3-bt), diluted in HPE. Unbound rhFHR-3-bt was washed away and the plates were incubated with 0.1% (v/v) streptavidin conjugated with HRP (strep-HRP, GE Healthcare) for 20 min. After washing, the assay was developed by addition of 100 μl of 100 μg/ml 3,5,3’,5’-Tetramethylbenzidine (TMB) in 0.11 M sodium acetate containing 0.003% (v/v) H_2_O_2_, pH 5.5. Substrate conversion was stopped after approximately 10 min by addition of 100 μl 2 M H_2_SO_4_. Absorbance was measured at 450 nm and corrected for the absorbance at 540 nm with a Synergy 2 Multi-Mode plate reader (BioTek Instruments, Winooski, VT, USA). All ELISA steps were performed with a volume of 100 μl per well. Positive wells were made monoclonal by multiple limiting dilution steps. Selected clones were allowed to grow and produce mAbs for up to 21 days prior to purifying the mAbs from the supernatant using a ProtA sepharose column. Isotypes of mAbs were determined by ELISA or with the use of the Mouse mAb Isotyping Kit (Hycult Biotech, Uden, the Netherlands) according to the manufacturer’s instructions.

### Cross-reactivity ELISA

Cross-reactivity of obtained anti-FHR-3 monoclonal and polyclonal antibodies was determined by ELISA. Briefly, Nunc Maxisorp 96-wells microtiter plates (Invitrogen) were coated overnight at RT with 2 μg/ml purified anti-FHR-3 monoclonal or polyclonal Abs in 0.1 M carbonate-bicarbonate buffer, pH 9.6. After washing, 10 nM of biotinylated rhFHRs, FH, or a 6xHis-tagged control protein, diluted in HPE, was added and incubated for 1 h, followed by another washing step and incubation with 0.01% (v/v) strep-poly-HRP (Sanquin), in HPE. All ELISA steps were performed with a volume of 100 μl per well and the ELISA was developed as described above.

### *CFHR* gene copy number determination

For the multiplex ligation-dependent probe amplification (MLPA) method, the SALSA MLPA probemix P236-A3 ARMD mix-1 was used to genotype the *CFH-CFHR* gene region according to the manufacturer’s instructions (MRC-Holland, Amsterdam, the Netherlands). This kit was previously used and validated in several other studies [11,14,15,19–21].

### Immunoprecipitation

FHR-3 was immunoprecipitated from human healthy donor serum using monoclonal antibodies against FHR-3. Immunoprecipitation (IP) was performed by incubating 200 μl serum with 500 μl of 5 mg/ml CNBr-activated Sepharose (GE Healthcare) to which RM-19 was coupled (25 mg mAb per 1 gram sepharose), and 50 μl of 100 μg/ml anti-FHR-3 mAb, diluted in PBS supplemented with 0.1% Tween-20, 0,1% BSA and 10 mM EDTA. Following overnight incubation at 4°C, while rotating, the sepharose was washed 3 times with 1 ml PBS + 0.02% (w/v) Tween-20 (PT) and 2 times with 1 ml PBS. Precipitated proteins were eluted by addition of 50 μl 1x NuPAGE Sample buffer solution (Invitrogen) and incubation at 70°C for 10 minutes. After spinning down the sepharose, SDS-PAGE under non-reducing conditions was performed using a Novex NuPAGE 10% Bis-Tris gel followed by Western Blot onto a nitrocellulose membrane (Novex iBlot Gel Transfer kit, Invitrogen). Membranes were blocked with 1% (v/v) Western Blocking Reagent (WBR, Roche, Basel, Switzerland) in PBS for 30 minutes and incubated with 1 μg/ml of either biotinylated polyclonal rabbit anti-FHR-3 or anti-FHR-3.1-bt in PBS + 0.5% (v/v) WBR, O/N. After washing three times with PT, membranes were incubated with 0.1% (v/v) Strep-HRP in PBS + 0.5% (v/v) WBR. After 1 hour, the membranes were washed three times with PT followed by two washes with PBS. Western Blots were developed with the Pierce ECL 2 Western Blotting substrate kit (Thermo Scientific) according to the manufacturer’s instructions and analyzed using the ChemiDoc MP System (BioRad, Hercules, CA, USA).

### Depletion of FHR-3 from human plasma

FHR-3 was depleted from normal human plasma using anti-FHR-3.1 or anti-FHR-3.4. Plasma was run over a 3 ml column containing either anti-FHR-3.1 or anti-FHR-3.4 coupled to CNBr-activated Sepharose 4B (GE Healthcare, 10 mg mAb per 1 gram sepharose coupled, according to the manufacturer’s instructions). Flowthrough was collected and used for IP using polyclonal anti-FHR-3 and ProtG-coupled sepharose (GE Healthcare) as described above.

### FHR-3 ELISA

To measure FHR-3 in serum, anti-FHR-3.1 was coated (2 μg/ml concentration in 0.1 M carbonate-bicarbonate buffer, pH 9.6) onto Nunc Maxisorp 96-wells microtiter plates (Invitrogen) by O/N incubation at RT. After washing, samples, diluted in HPE, were added and incubated for 1 hour at RT. Following washing with PT, 0.5 μg/ml biotinylated anti-FHR-3.4 (in HPE) was incubated on the plate for 1 hour at RT. The ELISA was developed using 0.01% (v/v) strep-poly-HRP (Sanquin) and TMB solution as described above. All steps were performed with a volume of 100 μl per well.

### FH ELISA

To measure FH in serum, anti-FH.16, directed against SCR 16/17, was coated (1 μg/ml concentration in PBS) onto Nunc Maxisorp 96-wells microtiter plates (Invitrogen) by incubating at RT, O/N. After washing, samples were diluted in HPE, added and incubated for 1 hour at RT. After another wash step, 0.25 μg/ml HRP-conjugated polyclonal goat anti-human FH was incubated on the plate for 1 hour at RT. Following washing, the ELISA was developed using TMB as described above. All steps were performed with a volume of 100 μl per well. FH levels in healthy donor sera were calculated using a serum pool of 400 donors containing 288 μg/ml FH.

### Statistics

Data were analyzed and all statistical tests were performed using GraphPad Prism, version 6.04 (GraphPad Software, La Jolla, CA, USA). Wilcoxon, Mann-Whitney and Kruskal-Wallis tests were used as indicated to assess significant differences. Correlation between FHR-3 and FH or CRP was assessed using a nonparametric Spearman’s correlation test.

## Results

### Generation of monoclonal and polyclonal antibodies against FHR-3

**Fig 1:**
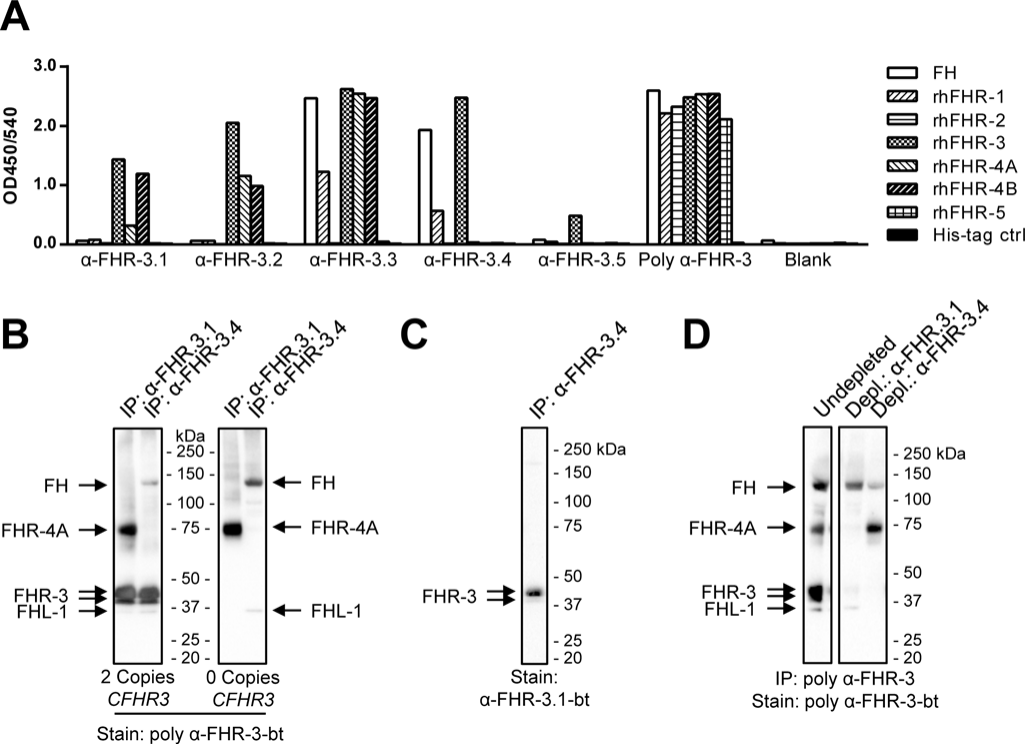
Characterisation of anti-FHR-3 mAbs. (A) Cross-reactivity of the obtained anti-FHR-3 monoclonal and polyclonal Abs. Wells coated with the indicated Abs were incubated with 10 nM of biotinylated FH, rhFHR proteins or an irrelevant 6xHis-tagged protein as control. Binding was determined by ELISA. (B) IP of FHR-3 from serum by anti-FHR-3.1 and anti-FHR-3.4 mAbs, visualized by Western Blot using poly anti-FHR-3-bt. Left panel; IP from healthy donor serum with two *CFHR3* gene copies. Right panel; IP from healthy donor serum with no *CFHR3* gene copies present (determined by MLPA). (C) IP of FHR-3 with anti-FHR-3.4 from a *CFHR3* sufficient donor, stained with anti-FHR-3.1-bt. (D) IP and staining of FH, FHR-3, FHR-4A and FHL-1 on Western blot from healthy donor plasma using poly anti-FHR-3, prior or after depletion with either anti-FHR3.1 or anti-FHR-3.4.

Five mAbs directed against rhFHR-3 were obtained and named anti-FHR-3.1 to anti-FHR-3.5. All mAbs were identified to be IgG1 and none of the mAbs competed with each other for the binding of FHR-3 (data not shown) indicating non-overlapping epitopes. All anti-FHR-3 mAbs were able to bind rhFHR-3 but most showed, although to a different extent, cross-reactivity with other members of the FH protein family (Fig 1A). Two anti-FHR-3 mAbs (3.1 and 3.2) cross-reacted with rhFHR-4A and rhFHR-4B. Anti-FHR-3.3 cross-reacted with FH, rhFHR-1, rhFHR-4A and rhFHR-4B and anti-FHR-3.4 cross-reacted with FH and FHR-1. Anti-FHR-3.5 was monospecific for FHR-3 but also had a relatively low-binding affinity and was not pursued further. The obtained rabbit polyclonal anti-FHR-3 was able to bind all of the rhFHR proteins as well as FH.

### *CFHR3* and *CFHR1* copy number variation in healthy donors

Specificity of the mAbs would be confirmed best by testing individuals with a homozygous deletion of the *CFHR3* gene. We assessed the CNV of *CFHR3* and *CFHR1* in Dutch healthy control individuals by MLPA (Table 1). In 67 of the 100 donors tested, no deletion or duplication events were detected. While 25 donors carried a heterozygous deletion of the *CFHR3*/*CFHR1* gene segment, four donors exhibited homozygous deletions of *CFHR3/CFHR1*. Two donors contained only one gene copy of either *CFHR3* or *CFHR1* while the other gene had two copies. Duplication events were detected in 2 donors, with 1 donor having 3 gene copies of *CFHR1* and the other donor having 3 gene copies of both *CFHR3* and *CFHR1*.

**Table 1:** *CFHR3/CFHR1* copy number variation in 100 healthy donors.

	*CFHR3*	*CFHR1*	*n*
**Gene copies**	2	2	67
*Deletion events*			
	0	0	4
	1	1	25
	1	2	1
	2	1	1
*Duplication events*			
	2	3	1
	3	3	1
			**Total:** 100

### Characterization of monoclonal antibodies against FHR-3

In order to specifically detect FHR-3 a combination of mAbs should be used that only share reactivity towards FHR-3. Therefor anti-FHR-3.1 and anti-FHR-3.4 were further characterized as combinations of other mAbs could potentially results in detection of other FH protein family members besides FHR-3, or be hampered by the presence of high levels of FH.

First, reactivity of anti-FHR-3.1 and anti-FHR-3.4 against human derived FHR proteins was confirmed by IP using normal healthy donor serum with two *CFHR3* gene copies (Fig 1B, left panel). Both mAbs immunoprecipitated proteins within the reported molecular mass of FHR-3 (37–50 kDa) when visualized by Western blot, run under non-reducing conditions and stained with the polyclonal anti-FHR-3 Abs. Cross-reactivity of anti-FHR-3.1 against FHR-4A and of anti-FHR-3.4 against FH, as previously determined by ELISA, was also confirmed by Western blot. We were neither able to detect FHR-4B nor FHR-1 from serum as would be expected with anti-FHR-3.1 and anti-FHR-3.4, respectively. However, FHL-1 was detected in the IP performed with anti-FHR3.4.

The pronounced bands at 37–50 kDa were absent when serum from a healthy donor without *CFHR3* gene copies was used (Fig 1B, right panel), confirming the specific reactivity of anti-FHR-3.1 and anti-FHR-3.4 against serum FHR-3. More importantly, IP of FHR-3 from a *CFHR3* sufficient donor with the anti-FHR-3.4 mAb, followed by detection on blot with the anti-FHR-3.1-bt mAb, only visualized bands consistent with the reported size of FHR-3 (Fig 1C).

To address whether all FHR-3 present in human was recognized by anti-FHR-3.1 and anti-FHR-3.4 we performed depletion experiments followed by IP and Western Blot using the polyclonal FHR-3 Abs. Indeed, FHR-3 could be completely depleted from human plasma using either anti-FHR-3.1 or anti-FHR-3.4 (Fig 1D), indicating that all FHR-3 is captured by these particular mAbs. Together, these results confirmed that this particular combination of anti-FHR-3 mAbs is suitable to determine FHR-3 serum levels in an ELISA without any cross-reactivity with other members from the FH protein family.

### Measuring FHR-3 specifically by ELISA using two monoclonal antibodies

As FH is an abundant protein in human serum, using anti-FHR-3.4, which cross-reacts with FH, as a catching mAb in a FHR-3 ELISA would likely result in interference of FH and possibly decreasing the sensitivity. Therefore anti-FHR-3.1, which does not cross-react with FH, was used as catching antibody and biotinylated anti-FHR-3.4 was used as detecting antibody. This allows for direct measurement of FHR-3 in human serum avoiding cross-reactivity with FH and hence does not require any depletion of FH prior to assessment of the serum FHR-3 levels.

**Fig 2:**
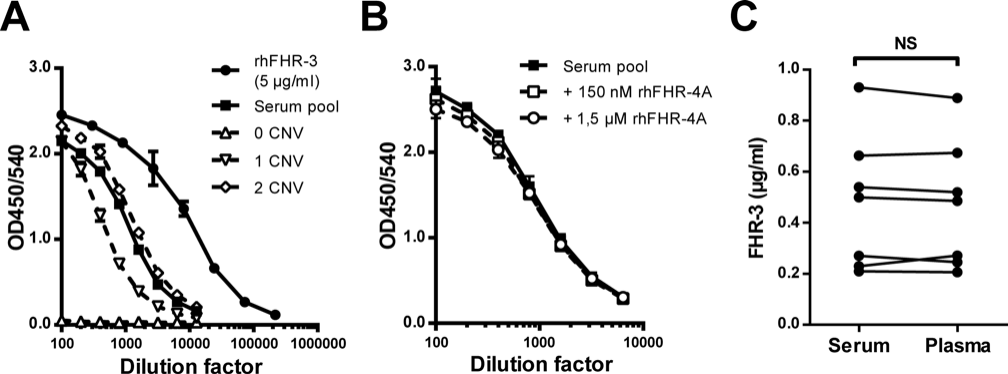
Characterisation of the FHR-3 ELISA with anti-FHR-3.1 and anti-FHR-3.4. (A) Titration of 5 μg/ml rhFHR-3, pool serum and serum of three donors with known copy numbers for *CFHR3* in the FHR-3 ELISA. Representative of three independent measurements, points represent the mean with error bars indicating SD of duplicates. (B) Influence of additional rhFHR-4A on FHR-3 levels measured in the FHR-3 ELISA. Pooled serum was incubated with excess rhFHR-4A, as indicated, prior to measuring the FHR-3 levels in the FHR-3 ELISA. Representative of two independent measurements, points represent the mean with error bars indicating SD of duplicates. (C) FHR-3 levels in serum and plasma as measured with the FHR-3 ELISA in 7 healthy donors. Points represent means of three independent measurements per donor. Wilcoxon paired test; NS = not significant.

Specificity of the ELISA was confirmed using serum from a healthy donor that has a homozygous deletion of *CFHR3/CFHR1* (determined by MLPA), as no signal was detected in this serum (Fig 2A). Furthermore, the curves of rhFHR-3 and pooled serum were parallel, indicating identical detection of FHR-3 in both serum and the recombinant sample. In addition, the donor with 1 CNV appeared to have lower FHR-3 in serum compared to the donor with 2 CNV. The serum pool was calibrated using highly pure rhFHR-3, of which the protein concentration was determined spectrophotometrically using an extinction coefficient of 17.83 (280 nm, 1% solution, incl. 6xHis-tag). After multiple independent measurements, the serum pool, comprised of more than 400 healthy donors, was determined to contain 0.554 μg/ml FHR-3, or 15 nM, and was used further on as calibration curve for the measurement of FHR-3 levels in a larger healthy donor cohort. As anti-FHR-3.1 cross-reacts with serum FHR-4A, we next assessed whether FHR-4A affects the measurement of FHR-3 by adding rhFHR-4A. As the physiological levels for FHR-4A are yet to be determined we added a 10-fold and 100-fold molar excess of rhFHR-4A relative to the FHR-3 concentration to our serum pool. Even at a 100-fold molar excess of rhFHR-4A, which corresponds with a concentration of 1,5 μM, or 129 μg/ml FHR-4A, in undiluted serum, we did not detect any difference in FHR-3 measured, strongly indicating that FHR-4A does not affect our FHR-3 ELISA (Fig 2B). Furthermore, FHR-3 levels did not differ between plasma and serum of seven healthy donors (*p* = 0.4688, Fig 2C).

### FHR-3 serum levels correlate with *CFHR3* gene copies

**Fig 3:**
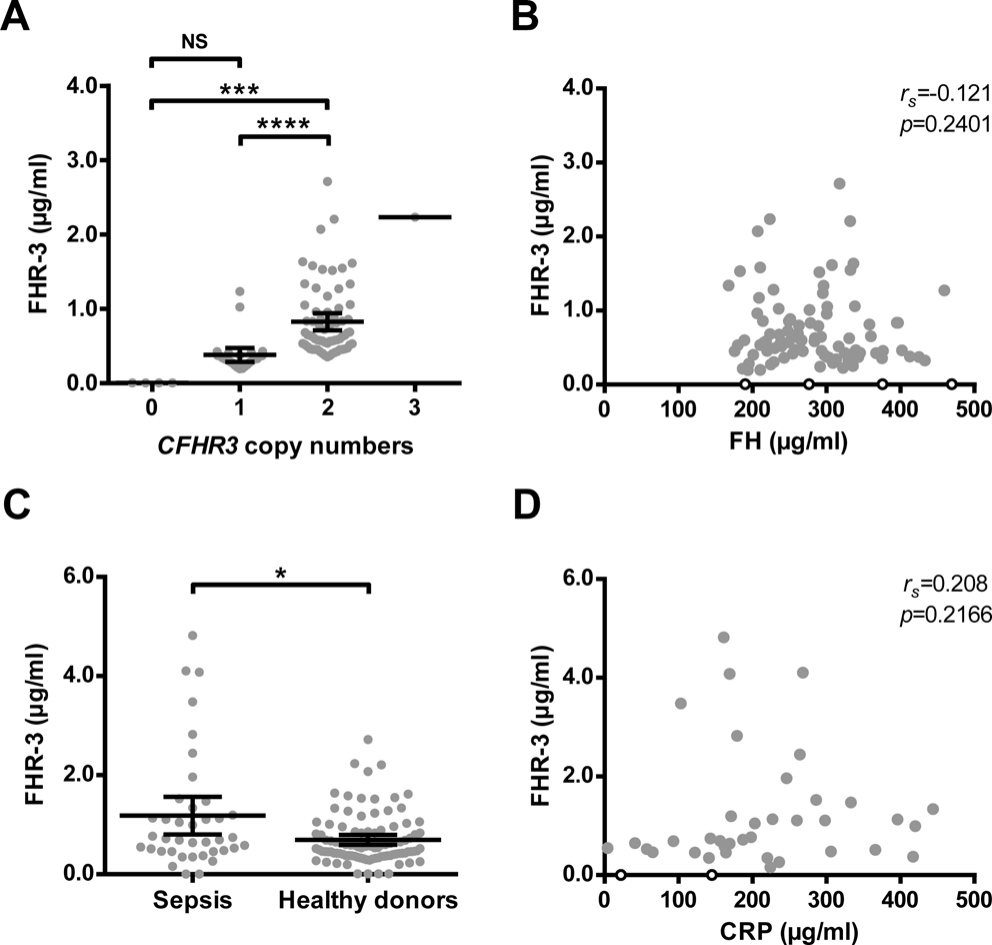
FHR-3 levels correlate with gene copy numbers and do not show an early acute phase response. (A) FHR-3 serum levels were measured by ELISA in 100 healthy donors and arranged by *CFHR3* gene copies as identified by MLPA. (B) FH serum levels were measured by ELISA in the same donors and depicted in relation to FHR-3 levels. The four donors with no copies of *CFHR3* (open symbols) were excluded from the correlation analysis (Spearman’s test). (C) FHR-3 levels were measured by ELISA in 39 acute sepsis patients and compared to FHR-3 levels of the healthy donor cohort depicted in A. (D) Graph depicting the correlation between FHR-3 levels and CRP levels. The two sepsis patients with apparent *CFHR3* deficiency (open symbols), as no FHR-3 was detectable, were excluded from the correlation analysis (Spearman’s test). Points in A-D represent the means of three independent measurements per donor or patient. Lines in A and C represents the mean with error bars indicating the 95% CI. Data were analyzed using the Kruskal-Wallis test (A) or Mann-Whitney test (B); NS = not significant, * *p*<0.05, *** *p*<0.001, **** *p<*0.0001.

With the use of the newly developed FHR-3 ELISA, FHR-3 serum levels were determined in 100 healthy donors of which we had determined the CNV of *CFHR3* by MLPA. FHR-3 levels significantly correlated with the presence of 1 or 2 *CFHR3* gene copies (Fig 3A).

Furthermore, no FHR-3 was detected in serum of healthy donors in which no *CFHR3* gene was detected, again proving the specificity of our assay. In the largest group of individuals, i.e. with 2 copies of the *CFHR3* gene, the mean concentration of FHR-3 was 0.83 μg/ml with a SD of 0.48 μg/ml, or 22.4 nM with a SD of 13 nM (considering a mass of 37 kDa). In individuals with only one gene copy present in their genome, FHR-3 levels were significantly lower (*p*<0.001) with a mean of 0.38 μg/ml and a SD of 0.23 μg/ml, or 10.3 nM (SD = 6.3 nM). Strikingly, FHR-3 levels in individuals with one *CFHR3* copy were approximately 50% lower compared to the FHR-3 levels in individuals with two gene copies. In the only individual in whom 3 copies of *CFHR3* were found, FHR-3 serum levels were relatively high with levels of about 2.3 μg/ml, but still within the normal range of the normal population carrying 2 copies of *CFHR3*.

FH serum levels assessed with our monospecific FH ELISA in the same 100 healthy donors were within the reported normal range of FH, with a mean of 284 μg/ml, or 1.8 μM, and a SD of 68 μg/ml (0.4 μM). There was no correlation with FHR-3 levels (*r*_*s*_ = -0.121, *p* = 0.2401, Fig 3B).

Having established the normal serum levels for FHR-3 to be lower than expected, we subsequently investigated whether FHR-3 levels increase as part of an acute-phase reaction during severe infectious disease by measuring FHR-3 levels in sepsis patients upon admission. The mean FHR-3 levels were significantly higher in the sepsis group (1.71-fold, *p* = 0.0173) compared to our healthy donor cohort (Fig 3C), but did not correlate with a classical marker for an acute phase reaction, i.e. CRP levels (*r*_*s*_ = 0.208, *p* = 0.2166, Fig 3D). Note that two apparent FHR-3-deficient patients were identified with our FHR-3 ELISA in this small cohort; these were not included in the correlation analysis.

## Discussion

We developed and validated the first FHR-3 specific ELISA to accurately measure the serum levels of FHR-3 in healthy donors as well as in severe disease. Unexpectedly, the serum FHR-3 levels in healthy individuals were much lower than the previously reported estimates and ranged from 0 to 2.7 μg/ml with a mean of 0.69 μg/ml (18.7 nM). Since gene copy numbers of *CFHR3* vary, our data on the serum levels of FHR-3 were related to the genotype of healthy individuals, demonstrating that the gene copy number in the individual’s genome determines to a very large extent their steady state serum levels. The mean concentration was 22 nM in donors with two *CFHR3* genes, whereas the FHR-3 levels were about 2-fold lower with a mean of 10 nM in donors with only one *CFHR3* gene, showing a strong relation between *CFHR3* copy numbers and FHR-3 serum levels. In our cohort of healthy controls, the *CFHR3/CFHR1* deletion occurred with a frequency of 17%, which is in concordance with previous reported frequencies on *CFHR3/CFHR1* CNV [11,13,15]. No FHR-3 could be detected in donors homozygous for the *CFHR3/CFHR1* deletion.

Our finding that the FHR-3 levels in healthy controls were 100-fold lower than the previously estimated values was remarkable. FHR-3 levels were previously estimated at 1–2 μM but the data and assay on which this estimation was based had never been formally described [15]. Based on our own results and the notorious cross-reactivity or lack of specificity of mAbs against FH and FHRs, it is conceivable that additional FH protein family members have affected previous estimations. As reported here for FHR-3, mAbs raised against FHR proteins commonly show cross-reactivity with other FHR proteins as well as FH due to the high sequence identity within this protein family. By using two anti-FHR-3 mAbs that only share their reactivity towards FHR-3, we were able to specifically distinguish FHR-3 from all other FH protein family members, resulting in establishing lower but accurate levels of FHR-3. This was confirmed by the lack of signal in healthy donors with a homozygous *CFHR3/CFHR1* deletion, demonstrating that our FHR-3 ELISA was effective in specifically detecting FHR-3.

However, the difference in the previously estimated levels of FHR-3 and the levels reported here might alternatively be attributed to an acute-phase response resulting in much higher levels of FHR-3 then measured during healthy conditions. Therefore, we measured FHR-3 in a small cohort of patients with sepsis. Although the mean FHR-3 levels were significantly increased in septic patients upon admission compared to healthy controls, this was only a 1.7-fold increase. Moreover, this increase seemed to be mainly caused by 6 patients in which we could not find any clear association with disease activity, pathogen or other parameters. A larger study in more homogeneous disease populations, which will include serum samples drawn after convalescence as well as establishing the corresponding CNV at the *CFH/CFHR* locus, is required to further investigate the higher FHR-3 levels associated with sepsis. We cannot formally exclude an acute phase protein response in some of the sepsis patients that we tested as we were not able to measure FHR-3 under healthy conditions in these patients and we had no DNA to verify the *CFHR3* CNV status in these individuals. On the other hand since we did not find any correlation of FHR-3 levels with CRP in samples drawn upon admission, our findings indicate that FHR-3 does not react as an early major acute phase protein, thus a major acute-phase response of FHR-3 does not account for the difference between the previously estimated FHR-3 levels and the levels reported here.

It is important to note that previous reports on functional characteristics of FHR-3 have used levels much higher than the physiological concentration of FHR-3 in human serum. For instance, Fritsche *et al*.(2010) demonstrated *in vitro* complement regulatory activity for FHR-3 at concentrations ranging from 10 up to 80 μg/ml [15]. Whether these reported functions are truly of physiological relevance remains to be elucidated but seems unlikely considering the much lower serum concentration of FHR-3. Furthermore, in the recently demonstrated *in vitro* competition between FH and FHR-3 for binding to meningococcal fHbp, Caesar *et al*. (2015) used up to a 10-fold molar excess of FHR-3 compared to FH [7], while *in vivo* the molar concentration of FHR-3 is on average 132-fold lower compared to FH in serum. In addition, the most common variant group of meningococcal fHbp, V1, was reported by the same group to demonstrate a ~20-fold lower binding affinity for FHR-3 compared to FH [7,22]. Nonetheless, our data could not formally exclude that the demonstrated competition between FHR-3 and FH in fHbp interactions could occur *in vivo* during meningococcal disease nor have we established local concentrations of FH and FHR-3 in tissues. Indeed, SNPs in both *CFH* and *CFHR3* are associated with altered susceptibility for meningococcal disease [5]. It is possible that the reported SNPs result in a higher expression and serum levels of FHR-3, affecting the competition of the protein with FH for binding to fHbp. To further investigate whether the proposed competition does occur during meningococcal infection, FH and FHR-3 levels should be longitudinally determined in genotyped patients throughout the disease episode and—preferably—upon reconvalesence. Such prospective studies are ongoing at the moment.

Several groups have shown that the *CFHR3/CFHR1* deletion is part of *CFH* haplotypes that are associated with decreased risk for AMD [23–26]. As these haplotypes also include SNPs in *CFH*, it has proven to be difficult to attribute the association to a specific SNP or to the *CFHR3/CFHR1* deletion itself. Some reports do indicate a direct link between the deletion and AMD [15,23]. Alternatively, the protective associations of the different haplotypes might also be explained by altered FH functionality and/or serum levels [25,27]. However, while MLPA is useful to determine single gene copy numbers or SNPs, it is unsuited to determine complete haplotypes. Therefore we could not determine in this study whether individuals with a single deletion of both *CFHR3* and *CFHR1* do indeed carry a haplotype previously associated with altered FH levels. Establishing FH, FHR-3 and FHR-1 levels for all different haplotypes associated with AMD is necessary to elucidate the underlying biological mechanism and the relative role for each of the proteins in AMD.

In conclusion, we described for the first time the accurate assessment of serum levels of FHR-3 in normal controls. We have demonstrated that the normal FHR-3 serum levels in humans are mainly determined by CNV of *CFHR3* and not by immediate early acute-phase responses upon serious infection. Since FHR-3 levels are about 130-fold lower compared to FH, our data indicate that the determination of serum levels for all FHR proteins in patients is a crucial step to support and further elucidate any genetic association and physiological relevance of the FHR proteins in various complement-related diseases.

## Abbreviations

AMD: age-related macular degeneration
CNV: Copy number variation
FH: complement factor H
fHbp: FH-binding protein
FHR-(1–5): FH-related protein 1 to 5
MLPA: Multiplex ligation-dependent probe amplification
